# Utilization of Municipal Healthcare Services Among High-Cost Older Patients in Norwegian Somatic Hospitals: A Cross-Sectional Registry Study

**DOI:** 10.1177/11786329251406082

**Published:** 2025-12-23

**Authors:** Morten Lønhaug-Næss, Monika Dybdahl Jakobsen, Bodil H. Blix, Karl Ove Hufthammer, Jill-Marit Moholt

**Affiliations:** 1 Department of Health and Care Sciences, Faculty of Health Sciences, UiT The Arctic University of Norway, Tromsø, Norway; 2 Department of Health and Care Sciences, Faculty of Health Sciences, Centre for Care Research North, UiT The Arctic University of Norway, Tromsø, Norway; 3 Faculty of Education, Arts and Sports, Western Norway University of Applied Sciences, Tromsø, Norway; 4 Centre for Care Research West, Western Norway University of Applied Sciences, Bergen, Norway; 5 Division of Rehabilitation Services, University Hospital of North Norway, Tromsø, Norway

**Keywords:** high-cost patients, older adults, municipal healthcare services, service utilization, somatic hospitals, Norway

## Abstract

**Background:**

High-cost older patients utilize a significant share of the economic resources in Norwegian somatic hospitals due to their complex healthcare needs and often require continued care beyond hospital settings. However, there is limited evidence on how they utilize municipal healthcare services.

**Objective:**

This study examines how high-cost older patients utilize municipal healthcare services, emphasizing variations across age categories, and comparisons with non-high-cost older patients.

**Design:**

Cross-sectional registry study using national registry data.

**Methods:**

The study population included 189 336 patients aged ⩾65 years with at least one unplanned contact with somatic hospitals in 2019, consisting of 18 933 (10%) high-cost and 170 403 (90%) non-high-cost older patients. High-cost status was defined as the top 10% of patients with the highest diagnosis-related group weights. Logistic regression was used to examine differences in the odds of receiving municipal healthcare services in the study population adjusted for individual characteristics. Among those who received services (n = 61 171), gamma regression was used to examine the duration of service use (hours/days). Both regression models were stratified by age categories (65-74, 75-84, ⩾85 years).

**Results:**

A higher proportion of high-cost than non-high-cost older patients received municipal healthcare services (54% vs 30%). This was also true after adjusting for patient characteristics (odds ratios 1.27-3.59, depending on age and type of healthcare service). High-cost patients had longer duration of service use (hours/days) than non-high-cost patients for institutional short-term treatment/examination (exp β = 1.12, *P* < .001) and rehabilitation/habilitation (exp β = 1.21, *P* < .001), but shorter duration for institutional long-term care (exp β = 0.80, *P* < .001).

**Conclusion:**

High-cost older patients have substantial healthcare needs that extend from somatic hospitals into municipal healthcare, highlighting the importance of such transitional services in caring for these patients.

## Introduction

In recent years, there has been an increasing attention on patients with complex healthcare needs due to concerns regarding health outcomes, service allocation, and the sustainability of healthcare systems.^1,2^ While various terms are used to describe these patients, in the context of somatic hospitals, high-cost patients are typically defined as those who utilize the most economic resources.^3-5^ Most of these are older adults who experience frequent acute hospital visits and prolonged admissions due to chronic conditions, multimorbidity, and frailty. Their complex healthcare needs frequently necessitate ongoing care beyond acute hospital treatment, extending into community and municipal healthcare services.^6,7^ Despite efforts to integrate healthcare across sectors, many high-cost older patients still face unmet healthcare needs.^2,8^ Research suggests that structural challenges such as insufficient continuity of care and poor integration during transitions from hospitals to municipal services may lead to higher rates of hospitalization.^9-12^ Other studies suggest that while the existing municipal healthcare systems might adequately meet the needs of many older adults, due to standardized service delivery, they may be inadequate in addressing the needs of those with complex and diverse health challenges.^13-15^ Consequently, many patients remain at home with substantial care needs, increasing their risk of acute hospitalization and reliance on specialized care.^16,17^

In Norway, there are no specific strategies directly focused on high-cost older patients,^3,18^ despite reports showing that less than 10% of patients, primarily older adults, account for more than 50% of costs in somatic hospitals, as estimated by diagnosis-related group (DRG) weights.^19^ However, health policies have sought to reduce avoidable hospitalizations and promote municipal healthcare services aimed at the older adult population. The 2012 Coordination Reform aimed to strengthen municipal healthcare capacity and improve cooperation between hospitals and municipalities,^20,21^ while the recently launched National Health and Collaboration Plan 2024 to 2027 aims to build on this effort to improve the coordination of care, particularly for patients with complex healthcare needs.^22^ Despite these initiatives, there is still limited knowledge about how high-cost older patients utilize municipal healthcare services in Norway. Existing research predominantly focuses on cost drivers (eg, multimorbidity) and individual patient characteristics.^23,24^ In the Nordic context, research has often focused on the general older adult population, relied on aggregated data, been confined to narrow geographical regions, or been conducted prior to the implemented reforms.^25-27^

Furthermore, research indicates that younger older adults often benefit from home healthcare services as they generally have fewer chronic conditions, and hospital admissions are often related to acute episodes.^28,29^ In contrast, transitional institutional care services are often essential for maintaining independence among the oldest patients following hospital discharge to reduce the likelihood of acute hospital readmissions.^27,30^ These services aim to support the short- and long-term healthcare needs of older adults through rehabilitation, chronic disease management, and assistance following hospitalization.^31,32^ Studies on high-cost populations rarely examine variations in service use across age categories, often treating high-cost older patients as a homogeneous group.^4^ As the use of healthcare services is known to vary across older age categories,^33^ stratification can help distinguish age-related differences from other factors driving healthcare utilization.^34^

### Norwegian Healthcare Services

The Norwegian public healthcare system is designed to provide comprehensive access to essential health services for all residents. Specialized medical services, such as surgery and treatment for severe illnesses, are centralized in hospitals managed by government-owned Regional Health Authorities.^35^ In contrast, primary care is decentralized to the municipalities, which are responsible for these services.^36^ Ideally, hospitals and municipal healthcare services should collaborate closely to ensure continuity of care after hospital discharge.^22,37-39^ However, research indicates that there are persistent challenges in coordination, with fragmented patient care pathways and gaps in transitions between healthcare levels.^25,40^

At the primary level, municipalities are tasked with providing a wide range of healthcare services, including both home healthcare, institutional care, and out-of-hours emergency services.^36^ Norway has a well-established system for financing municipal healthcare services where home healthcare services are covered in its entirety by the public funding, while institutional short-term and long-term care requires out-of-pocked user contributions.^41^ Priority setting in municipalities are subject to several types of laws and regulations; its main principle is a utilitarian cost/effectiveness calculation weighted for severity,^39,42^ where service allocation is intended to follow the “Best Effective Care Level” (BEON) principle.^32^ The intention is to provide less complex and costly services, such as home healthcare, before turning to institutional care.^32,43,44^ However, it is worth noting that different considerations, such as cost-saving, and local conditions, such as resource availability in municipal priority settings, may lead to indeterminacy in decision-making, affecting how local services are allocated.^42,45^ In addition, earlier hospital discharges, driven by policies that shift responsibilities from hospitals to municipalities, have placed pressure on municipal services often resulting in challenges for municipalities to provide adequate follow-up care.^21,46^ Despite efforts to improve coordination and continuity of care, municipalities sometimes lack the capacity to meet the rising demands for post-hospitalization care, leading to gaps in service delivery.^47^

This study aims to examine the use of municipal healthcare services among high-cost older patients emphasizing variations in service utilization across age categories and comparisons with non-high-cost older patients. Specifically, this study focuses on the utilization of home healthcare and institutional short-term and long-term care services. The research questions (RQ) guiding this study are as follows:

**RQ I**: What proportions of high-cost and non-high-cost older patients receive home healthcare and institutional municipal healthcare services, and are there differences in the probability of receiving these services between high-cost and non-high-cost patients also when adjusted for individual characteristics?
**RQ II**: Among those who do receive municipal healthcare services, how does the duration of services (measured in hours/days) differ between high-cost and non-high-cost older patients?
**RQ III**: How do the high-cost versus non-high-cost patients’ patterns in service utilization (RQ I and RQ II) vary across age categories?

## Materials and Method

### Study Design and Data Sources

This study is an observational cross-sectional study utilizing registry data from three national registries. The Norwegian Patient Registry (NPR) captures specialized healthcare activity, including patient demographics, diagnoses, and DRG weights. The Norwegian Registry for Primary Health Care (NRPHC) includes nursing and care statistics (IPLOS), documenting the type and amounts of municipal services received. Statistics Norway (SSB) provides sociodemographic data, such as income, education, marital status, and municipal centrality index.

### Study Population and Inclusion/Exclusion Steps

The study is part of a larger research initiative examining the use of municipal healthcare services among older adults with acute hospital contacts. In line with common practices in gerontological and health services research, we defined older adults as individuals aged 65 years and older.^48^ As the study aimed to include the entire population of individuals aged 65 years and older who had at least one acute contact with somatic hospitals in Norway from January 1st to December 31st, 2019 (n = 212 020), no power calculation was used to determine the sample size. Acute contacts are defined as unplanned/acute day-treatments, outpatient care, or hospital admissions involving an acute incidence.^49^ We excluded patients who opted out of having their data used for research purposes (n = 95) and patients with missing DRG information (n = 189). Approximately 11% (n = 22 402) of the study population died during 2019. These individuals were excluded from the main analyses to ensure a complete dataset on municipal healthcare utilization, as the estimates of their service utilization potentially could be incomplete or misleading. The sample size (n = 189 336) reflects the total eligible study population after applying the exclusion criteria.

### High-Cost Status

We defined high-cost status by creating a binary variable consisting of high-cost and non-high-cost older patients in Norwegian somatic hospitals using DRG weights as a proxy measure for costs. This dichotomous classification is commonly used in health services research to identify and characterize high-cost populations.^4,5,50^ The DRG system gathers detailed and standardized data about the in-hospital services patients receive, playing a key role in reimbursement provider payment.^51^ It calculates costs based on factors such as patient characteristics, procedures performed, severity of diagnoses, and type of admission.^52^ DRG weights are used to represent the relative costs associated with a hospital stay within a specific DRG group compared to the national average, where 1.0 reflects the average cost per patient.^53^ We defined the top 10% (90th percentile) as the high-cost group, corresponding to a cumulative DRG weight of 5.519 and higher. This group consisted of 18 933 patients, and the remaining 170 403 (90%) were classified as belonging to the non-high-cost group. The high-cost group had a mean DRG weight of 9.48 while the non-high-cost group had a mean of 1.45. In 2019, Norwegian somatic hospitals collectively produced approximately 1 539 771 DRG points,^54^ each valued as 44 654 NOK (4047 USD),^53,55^ amounting to an estimated national hospital expenditure of 67 billion NOK.^56^ While DRG weights do not equal actual incurred costs at the individual level, they provide a standardized proxy for relative resource use.^57^ We translated DRG weights into estimated NOK using the national average reimbursement per DRG to illustrate the scale of hospital resource consumption.^53^ Based on this estimate, the high-cost group’s accumulated DRG weights correspond to at least five and half times the average hospital costs per patient.

### Individual Characteristics Affecting Healthcare Utilization

To account for individual characteristics that influence healthcare utilization and the use of municipal services, we included the following variables in our regression model: household income, educational level, marital status, and index of municipal centrality. These variables were obtained from SSB, and chosen based on existing literature,^24,58-60^ and insights from a prior study.^50^ The recording, coding, and detailed rationale for including the characteristics are presented in Supplemental Table 1. Household income is the sum of all individual incomes of the household members and is recorded in six income level categories, but the information was only available for individuals alive at the end of 2019. Educational level was recorded in five categories and recoded into four variables as primary school, high school/vocational school, higher education up to 3 years, and higher education 4 years and beyond. Marital status was recorded in four categories and recoded into two categories as either single/unmarried/separated/divorced/widowed or married/registered partner/cohabitant. The index of municipal centrality compares the centrality of entire municipalities relative to each other and was recorded using six categories ranging from the least to most central municipalities. We also included the number of different registered main diagnoses a patient was registered with as a proxy measure for multimorbidity. This characteristic was constructed based on the 22 various main diagnoses from NPR.

### Municipal Healthcare Services

We included statutory services,^31,32^ which represent municipal healthcare at different levels of care. The services included are as follows:

*Home healthcare services*: Refer to all forms of planned municipal health services provided in the patient’s home or starting from the patient’s home.^61^ These services are provided to individuals needing support due to illness or disability, and which home healthcare services are provided depends on the patient’s care needs.^32^ Examples are home-care nursing, occupational therapy, and physiotherapy. Home-based services which do not involve healthcare, such as domestic care and personal assistance, are excluded from this study.
*Institutional short-term care*: We included three types of services relevant as intermediate care before or after hospitalization: (1) Municipal acute bed units offer round-the-clock care for a time-limited period (usually maximum 72 hours) for patients requiring immediate medical attention where hospitalization otherwise would be needed.^62,63^ Acute bed units also admit patients after hospital discharge to optimize treatment before being transferred to their home or municipal care facilities/nursing homes. (2) Short-term care for treatment/examination purposes are granted during an ongoing illness or for clarification on future care provision. (3) Rehabilitation/habilitation is granted to increase the patient’s independence, physical and functional abilities, supporting recovery and functional improvement following illness or injury. Short-term care for treatment or rehabilitation takes place in designated municipal facilities and are time-limited for up to 60 days each calendar year with an overarching goal for the patient to return to their home or other place of residence after completed treatment or rehabilitation.^43^

*Institutional long-term care*: Refers to a stay in a nursing home or similar care facility that provides round-the-clock care of indefinite duration or exceeding 60 days within a calendar year. This service is offered to those who have permanent health problems and extensive need for nursing care and medical treatment which cannot be addressed at home, and where institutional care is the only offer that can ensure the provision of proper services.^38,64^

### Measurement of Municipal Healthcare Services

Home healthcare services were measured in hours, while all institutional care services were recorded as the total number of days during 2019. If a patient had multiple entries for the same type of service, these entries are treated by NRPHC as separate periods, regardless of whether they overlapped in time. This is because municipalities operate with a more nuanced service coding system in their journal systems than what is reported to NRPHC. As a result, the number of hours/days a person was recorded to receive the service in 2019 could exceed 8760 hours (12 months)/365 days.^65^ This discrepancy likely occurs when a new service is registered before the previous one is formally terminated, leading to overlapping registrations.

### Age Stratification

To explore variations in service use across age, patients were divided into three age categories: 65 to 74 years (n = 83 195), 75 to 84 years (n = 67 436), and 85 years and older (n = 38 705). This stratification aligns with prior research emphasizing age-related differences in healthcare utilization.^66,67^

### Statistical Analysis

To examine the proportions of those in the high-cost and non-high-cost groups who received home healthcare and institutional municipal services (*RQ I*), we report numbers and proportions of individuals in the respective groups and compared the groups using Pearson’s chi-squared test. A binary logistic regression analysis model was used to compare the odds of receiving municipal healthcare services in the high-cost and non-high-cost groups adjusted for sex, marital status, educational level, household income, index of municipal centrality and number of main diagnoses. Adjusting for these factors allowed us to isolate the effect of being in the high-cost or non-high-cost group on service utilization while accounting for individual characteristics that might otherwise confound the results. We report the results as odds ratios (OR), where an OR > 1 indicates that a patient in the high-cost group had a higher estimated probability of receiving the given municipal healthcare service compared to a patient in the non-high-cost group with similar individual characteristics. An OR < 1 suggests a lower probability.

To investigate how the duration (hours/days) of services received differed between the high-cost and non-high-cost groups (*RQ II*), we report the mean duration when including only the participants who received each service. The difference in mean duration between the two groups was compared using Welch’s *t*-test. This test was chosen due to different variances in the two groups and because the non-high-cost group was nine times as large as the high-cost group. To further account for differences in characteristics between the two groups, we fitted gamma regression models with a log link and robust (Huber-White) covariance estimation to model the ‘expected’ number of hours or days of services received. The model was adjusted for the same characteristics as the logistic regression model, and we report the exponentiated coefficient, exp β, for being in the high-cost group (versus the non-high-cost group). An exp β > 1 represents a proportional higher expected total number of days or hours. For example, an exp β of 1.30 equals 30% higher expected total number of hours or days for a patient in the high-cost group than for the non-high-cost group with similar characteristics, while 0.7 represents 30% lower expected total number of hours or days.

We stratified all descriptive statistics and regression analyses by the age categories: 65 to 74, 75 to 84, and 85 years and older (*RQ III*). In the final study sample, there were 2714 patients (1.4%) with missing data on one or more characteristics such as household income, educational level, marital status, or index of municipality centrality (205 in the high-cost group and 2509 in the non-high-cost group). Given the minimal level of missing data, we opted for complete-case analyses in all regression models. We set the significance level at .05, all confidence intervals (CIs) reported are 95% CI, and all analyses were performed using SPSS version 29.0.1.1. or R version 4.3.1/4.3.2.

### Sensitivity Analysis

We performed two sensitivity analyses. First, to account for possible data artifacts from overlapping service registrations, we excluded participants whose service use exceeded 1 year (8760 hours/365 days). As our dataset does not include information regarding start and end dates for these services, we were unable to verify whether such overlap represents concurrent service use or registration discrepancies. In the second analysis, patients who died in 2019 (and who were excluded in the main analysis due to incomplete data) were included to evaluate whether their exclusions influenced the results. This allowed us to determine if mortality-related healthcare utilization affected the observed trends in service use among the high-cost and non-high-cost groups.

## Results

### Distribution of Individual Characteristics

The distribution of individual characteristics between the high-cost and non-high-cost groups is presented in Table 1. Men make up a larger proportion of the high-cost group (56%) compared to the non-high-cost group (46%). The high-cost group also has a slightly younger age distribution, with 47% aged 65 to 74 years versus 44% in the non-high-cost group, while a higher proportion of the non-high-cost group is aged 85 years and older (21% vs 14%). Marital status, income levels, educational attainment, and municipal centrality are more similarly distributed between the groups, with some minor variations. However, the high-cost group has a substantially higher prevalence of multiple main diagnoses, with 33% having five or more diagnoses compared to only 6% in the non-high-cost group.

**Table 1.** Overview of the Included Individual Characteristics Between the High-cost and Non-High-Cost Groups (n = 189 336).

Individual characteristics	High-cost group (n = 18 933; n (%))	Non-high-cost group (n = 170 403; n (%))
*Sex*		
Men	10 506 (56)	77 814 (46)
Women	8427 (44)	92 589 (54)
*Age*		
65-74 years	8952 (47)	74 243 (44)
75-84 years	7266 (39)	60 170 (35)
⩾ 85 years	2715 (14)	35 990 (21)
*Marital status*		
Single, separated, divorced, widowed	10 328 (55)	87 267 (51)
Married, cohabitant	8605 (45)	83 136 (49)
*Educational level*		
Primary school	5827 (31)	50 646 (30)
High school/vocational school	9336 (49)	81 502 (48)
University/college short	2646 (14)	27 048 (16)
University/college long	940 (6)	9092 (5)
*Household income*		
<150 000 NOK	84 (<1)	817 (1)
150-250 000 NOK	2375 (12)	26 387 (15)
251-350 000 NOK	3590 (19)	31 892 (19)
351-450 000 NOK	2201 (12)	19 662 (11)
451-550 000 NOK	3527 (19)	28 642 (17)
551-750 000 NOK	4407 (23)	37 059 (22)
751-1 000 000 NOK	1634 (9)	14 890 (9)
>1 000 000 NOK	1104 (6)	10 530 (6 )
*Index of municipal centrality*		
Most central municipalities	2868 (15)	26 843 (16)
Second most central municipalities	4428 (23)	40 121 (24)
Mid-central I municipalities	4723 (25)	42 565 (25)
Mid-central II municipalities	3369 (18)	30 557 (18)
Second least central municipalities	2290 (12)	19 235 (11)
Least central municipalities	1234 (7)	10 455 (6)
*Main diagnosis groups*		
1 main diagnosis	1349 (7)	58 558 (34)
2 different main diagnoses	3140 (16)	52 604 (31)
3 different main diagnoses	4306 (23)	32 191 (19)
4 different main diagnoses	3926 (21)	16 217 (10)
5 or more different main diagnoses	6212 (33)	10 833 (6)

Missing information – high-cost group: Educational attainment (n = 184), Household income (n = 11), Index of municipal centrality (n = 21) – non-high-cost group: Educational level (n = 2229), Household income (n = 524), Index of municipal centrality (n = 627).

### Proportions and Odds Ratios of Utilizing Municipal Healthcare Services Among the High-cost and Non-High-Cost Groups (RQ I)

The number and proportion of participants in the high-cost and non-high-cost groups who utilized municipal healthcare services in 2019 are presented in Table 2. In total, 61 171 participants (32%) in the study sample received one or more municipal healthcare services during 2019. Among the high-cost group, 54% (n = 10 304) received any municipal healthcare services, compared to 30% (n = 50 867) in the non-high-cost group (*P* < .001). Additionally, 23% (n = 4261) of the high-cost group received two or more different types of services, compared to 11% (n = 17 855) in the non-high-cost group (*P* < .001). The high-cost group also had higher proportions of individuals using each municipal service type across age categories than the non-high-cost group, particularly for home healthcare services (45% vs 23%), institutional short-term treatment/examination (16% vs 7%) and rehabilitation/habilitation care (13% vs 5%), all significant at *P* < .001. For municipal acute bed units (5% vs 4%, *P* < .001) and institutional long-term care (5% vs 5%) this difference was smaller, and not statistically significant for institutional long-term care (*P* = .73)

**Table 2.** Number and Proportion of the High-Cost and Non-High-Cost Groups in the Study Sample (n = 189 336) Who Received Municipal Healthcare Services in 2019, Stratified by Age.

Municipal healthcare services: n (%)	High-cost group (n = 18 933)	Non-high-cost group (n = 170 403)	*P*-value*
*Home healthcare services*			
65-74 years	3314 (37)	7897 (11)	<.001
75-84 years	3503 (48)	14 754 (25)	<.001
⩾85 years	1777 (66)	17 207 (48)	<.001
All age categories	8594 (45)	39 858 (23)	<.001
*Municipal acute unit*			
65-74 years	312 (4)	1061 (1)	<.001
75-84 years	400 (6)	2106 (4)	<.001
⩾85 years	295 (11)	3078 (9)	<.001
All age categories	1007 (5)	6245 (4)	<.001
*Institutional short-term care: treatment/examination*			
65-74 years	919 (10)	1844 (3)	<.001
75-84 years	1308 (18)	4305 (7)	<.001
⩾85 years	886 (33)	6468 (18)	<.001
All age categories	3113 (16)	12 617 (7)	<.001
*Institutional short-term care: rehabilitation/habilitation*			
65-74 years	768 (9)	1437 (2)	<.001
75-84 years	1029 (14)	2754 (5)	<.001
⩾85 years	629 (23)	3449 (10)	<.001
All age categories	2426 (13)	7640 (5)	<.001
*Institutional long-term care*			
65-74 years	184 (2)	829 (1)	<.001
75-84 years	349 (5)	2500 (4)	.009
⩾85 years	376 (14)	4766 (13)	.364
All age categories	909 (5)	8095 (5)	.731
*Received any municipal healthcare services* **			
65-74 years	3927 (44)	9974 (13)	<.001
75-84 years	4213 (58)	18 684 (31)	<.001
⩾85 years	2164 (80)	22 209 (62)	<.001
All age categories	10 304 (54)	50 867 (30)	<.001
*Received two or more different municipal healthcare services* ***			
65-74 years	1214 (14)	2474 (3)	<.001
75-84 years	1787 (25)	6013 (10)	<.001
⩾85 years	1260 (46)	9368 (26)	<.001
All age categories	4261 (23)	17 855 (11)	<.001

* Chi-squared test.

** Received any of the included municipal services: *home healthcare, municipal acute units, short-term care: treatment examination, short-term care: rehabilitation/habilitation, long-term care*.

*** Received two or more different services among the included services.

The adjusted odds ratio of the high-cost group receiving various municipal healthcare services across age categories, compared to the non-high-cost group with similar characteristics, is presented in Table 3. The corresponding unadjusted regression results are reported in Supplemental Table 2. The results indicate that being in the high-cost group is associated with higher odds of receiving municipal healthcare services across all service types, including institutional long-term care. For all age categories, the highest odds were observed for institutional short-term rehabilitation/habilitation (OR = 2.74, *P* < .001), home healthcare services (OR = 2.37, *P* < .001), and institutional short-term treatment/examination (OR = 2.23, *P* < .001).

**Table 3.** Logistic Regression Model for the Odds of Receiving the Various Municipal Healthcare Services for the High-Cost Group Compared to the Non-High-Cost Group in the Study Sample (n = 189 336), Adjusted for Individual Characteristics, and Stratified by Age.

Municipal healthcare services	OR	95 % CI	*P*
*Home healthcare services*			
65-74 years	3.59	3.39-3.79	<.001
75-84 years	2.38	2.26-2.52	<.001
⩾85 years	1.68	1.54-1.83	<.001
All age categories	2.37	2.29-2.45	<.001
*Municipal acute bed units*			
65-74 years	1.61	1.39-1.85	<.001
75-84 years	1.22	1.09-1.38	<.001
⩾85 years	1.03	0.90-1.18	.634
All age categories	1.17	1.08-1.26	<.001
*Institutional short-term care: treatment/examination*			
65-74 years	3.36	3.06-3.69	<.001
75-84 years	2.50	2.32-2.69	<.001
⩾85 years	1.91	1.74-2.09	<.001
All age categories	2.23	2.13-2.34	<.001
*Institutional short-term care: rehabilitation/habilitation*			
65-74 years	3.55	3.20-3.93	<.001
75-84 years	2.94	2.70-3.19	<.001
⩾85 years	2.48	2.23-2.75	<.001
All age categories	2.74	2.60-2.89	<.001
*Institutional long-term care*			
65-74 years	1.78	1.49-2.13	<.001
75-84 years	1.39	1.22-1.57	<.001
⩾85 years	1.32	1.17-1.49	<.001
All age categories	1.27	1.18-1.37	<.001

Abbreviations: CI, confidence interval; OR, odds ratio.

The model is adjusted for: Sex, marital status, educational level, household income, index of municipal centrality, and number of main diagnoses.

### Differences in Duration (Hours/Days) of Municipal Healthcare Services Among High-cost and Non-High-Cost Older Patients (RQ II)

The duration of municipal services utilized by the those in the high-cost and the non-high-cost group who *had* received each type of service, is detailed in Table 4. The supplementary descriptive and bivariate analyses, as well as the unadjusted regression results, are presented in Supplemental Tables 2 and 3, respectively. For all age categories, the high-cost group had significantly longer duration of service use than the non-high-cost group, for municipal acute bed units (exp β = 1.15, *P* = .09), institutional short-term treatment/examination (exp β = 1.12, *P* < .001), and rehabilitation/habilitation (exp β = 1.21, *P* < .001), but shorter duration of service use for institutional long-term care (exp β = 0.80, *P* < .001).

**Table 4.** Gamma Regression Model for the Duration (Hours/Days) of Municipal Healthcare Service Use Among the High-Cost Group Who Received Services Compared to the Non-High-Cost Group (n = 61 171), Adjusted for Individual Characteristics and Stratified by Age.

Municipal healthcare services	exp β	*P*-value	95% CI
*Home healthcare services* *			
65-74 years	0.88	.024	0.79-0.98
75-84 years	1.00	.966	0.93-1.08
⩾85 years	1.11	.021	1.02-1.21
All age categories	0.97	.226	0.92-1.02
*Municipal acute unit* **			
65-74 years	1.24	.036	1.01-1.51
75-84 years	1.16	.231	0.91-1.49
⩾85 years	1.05	.729	0.81-1.36
All age categories	1.15	.096	0.96-1.36
*Institutional short-term care: treatment/examination* **			
65-74 years	1.16	.004	1.05-1.28
75-84 years	1.06	.138	0.98-1.13
⩾85 years	1.21	<.001	1.12-1.30
All age categories	1.12	<.001	1.07-1.17
*Institutional short-term care: rehabilitation/habilitation* **			
65-74 years	1.16	.004	1.05-1.28
75-84 years	1.18	<.001	1.09-1.27
⩾85 years	1.30	<.001	1.20-1.42
All age categories	1.21	<.001	1.15-1.27
*Institutional long-term care* **			
65-74 years	0.77	<.001	0.67-0.87
75-84 years	0.78	<.001	0.71-0.85
⩾85 years	0.80	<.001	0.73-0.87
All age categories	0.80	<.001	0.76-0.84

Abbreviations: CI, confidence interval; exp β, exponentiated coefficient.

The model is adjusted for: Sex, marital status, educational level, household income, index of municipal centrality, and number of main diagnoses.

Gamma regression with log link for the duration (hours/days) of municipal healthcare service use among the high-cost and non-high-cost groups, stratified by age.

* Measured in hours of services received.

** Measured in days admitted. Reported coefficient for the high-cost group.

### Variations in Patterns of Municipal Healthcare Service Utilization Across Age Categories (RQ III)

For each age category, a larger proportion of the high-cost group than of the non-high-cost group used each service, with the exception for institutional long-term care among those aged 85 years and older (Table 2). We observed that those aged 85 years and older had the highest proportion of municipal service users. This variation was highest for home healthcare services and institutional short-term care. The smallest variation was observed for institutional long-term care, where the high-cost group only had 1 percentage point higher proportion of service users than the non-high-cost group for each of the age categories.

Across the various municipal healthcare services, we observed that also after adjusting for individual patient characteristics, the high-cost group consistently had higher odds for receiving services than the non-high-cost group for each of the age categories (Table 3). Compared to an individual in the non-high-cost group with similar characteristics, the highest odds were observed among those in the high-cost group aged 65 to 74 years for home healthcare services (OR = 3.59, *P* < .001), institutional short-term rehabilitation (OR = 3.55, *P* < .001) and treatment/examination (OR = 3.36, *P* < .001).

When examining the duration (hours/days) of service use (Table 4), we observed several variations. Compared to an individual in the non-high-cost group with similar characteristics, those in the high-cost group had longer duration of service use for institutional short-term care services, but shorter for institutional long-term care, for each of the age categories. Individuals in the high-cost group aged 65 to 74 years had shorter duration of home healthcare services compared to the non-high-cost group (exp β = 0.88, *P* = .024), whereas those aged 85 years and older had longer duration (exp β = 1.11, *P* = .021). For municipal acute bed units, those in the high-cost group aged 65 to 74 years had longer duration of service use than the non-high-cost group (exp β = 1.24, *P* = .036), while there was no significant association for the other age categories (*P* > .05).

### Sensitivity Analysis

To assess the impact of outliers, we performed regression analyses where we excluded the 349 living participants and 16 deceased participants who had service registrations exceeding 365 days or 8760 hours (about 12 months). We observed minor changes in the results, with trends and significance levels remaining consistent (Supplemental Table 4). We then included the 22 402 deceased participants in the regression models to determine if mortality-related healthcare utilization could have influenced our main results. The analysis confirmed that the findings remained consistent, with no substantial changes to the study’s conclusions (Supplemental Table 5).

## Discussion

### Main Findings

This study aimed to examine how high-cost older patients utilize municipal healthcare services, emphasizing variations across age categories, and comparisons with non-high-cost older patients. One main finding was that a larger proportion of the high-cost group utilized municipal healthcare services than the non-high-cost group in 2019, also after adjusting for individual characteristics (*RQ I*). Among those who received services, the duration (hours/days) of service use was longer for the high-cost group than the non-high-cost group for institutional short-term treatment/examination and rehabilitation/habilitation (*RQ II*). However, for institutional long-term care, the high-cost group had shorter duration of service use than the non-high-cost group. Lastly, we observed that the proportions of service use increased with age in both the high-cost and non-high-cost groups (*RQ III*). However, the duration of service use varied across age categories.

### High-Cost Older Patients Have Substantial Healthcare Needs That Extend from Somatic Hospitals into Municipal Healthcare Services

Approximately half of the high-cost group utilized municipal healthcare services in 2019, with nearly one-quarter using two or more service types, which suggests that the healthcare needs of high-cost older patients in Norway extend beyond somatic hospitals and into municipal care. The services that were most used by the high-cost group were home healthcare (45%) and institutional short-term treatment/examination (16%) and rehabilitation/habilitation (13%). This aligns with previous research indicating that older adults with complex healthcare needs, such as high-cost older patients, are more likely to receive municipal healthcare services such as home- and transitional care services,^68,69^ particularly following hospitalization. They may also require more support than other older adults after hospital discharge to facilitate recovery, manage long-term care needs, and reduce the risk of readmissions.^63,70,71^ Previous studies have associated prolonged municipal transitional care stays with extended hospital admissions and complex healthcare needs,^72-74^ due to longer time required for stabilization or rehabilitation following hospital discharge. This may also be evident in our study as we observed consistently higher odds of receiving municipal healthcare services among the high-cost group compared to the non-high-cost group. Additionally, the high-cost group had longer duration of use for institutional short-term care services. However, the shorter durations of institutional long-term care among the high-cost group warrant further investigation. It is challenging to interpret this observation within the context of our study and further examination would require other study designs which could provide a more nuanced understanding of service utilization in a longitudinal perspective.

### Variations in Municipal Healthcare Service Utilization Between High-cost and Non-High-Cost Older Patients and Across Age Categories

The observed variations in service utilization among the high-cost group remained significant after adjusting for individual characteristics and the number of main diagnoses. This suggests that the variations in service use cannot be attributed solely to the individual characteristics recorded, but to factors not captured by our study’s data, such as the severity of diagnoses and complex healthcare needs. The observed variation in healthcare service use further underscores that the service utilization among high-cost older patients is primarily driven by the complexity of healthcare needs, as indicated by prior studies.^4,75^ Thus, it is possible that the high-cost group may have more exacerbations, complications, or more advanced illness than those in the non-high-cost group with similar characteristics. According to a systematic review, the health issues among high-cost populations were predominantly attributed to severe and acute medical conditions.^4^ Previous research has identified the most prevalent main diagnoses among Norwegian high-cost older patients to affect the circulatory, respiratory, digestive, nervous, and musculoskeletal systems.^50^ Conditions within these main diagnoses are often associated with high use of healthcare services, especially when they coexist.^18,67^ Interestingly, the largest absolute and relative difference in service use between the high-cost and non-high-cost groups is observed in the 65 to 74 years age category. This observation may reflect the relatively greater heterogeneity in health status and care needs within this age category compared to those of older age, who may have progressed to advanced stages of illness and disability. This heterogeneity might arise because younger older adults are in a transitional period where some remain largely independent while others begin to require more support due to the onset of chronic illnesses.^76,77^ Early stages of multimorbidity in this age group often require more diverse healthcare interventions,^78^ which may explain the difference in municipal healthcare service utilization compared to that of the non-high-cost group in the same age category. A systematic review has previously examined the complexity and variability of care needs among individuals with multimorbidity, highlighting the importance of tailored and co-designed care approaches as standardized interventions often fail to address the diverse needs of this population.^79^ Proactive identification of older adults at risk of becoming future high-cost patients, and individualized care planning could improve outcomes and potentially moderate escalating healthcare needs before more severe disability or disease develops.^80,81^

### The Role of Municipal Healthcare Services in Caring for Older Adults with Complex Healthcare Needs

The results from our study should be interpreted within the context of Norwegian municipal healthcare, where the principle of providing care at the best effective level (BEON) aims to ensure that services are allocated to meet individual needs at the appropriate level of care.^32,43^ The high level of service utilization among the high-cost group may indicate municipal efforts to prioritize and allocate resources to those with the greatest needs.^32^ It appears that Norwegian municipal healthcare services are fulfilling their intended purpose,^22,38^ although local variations in allocation practices cannot be ruled out.^42,45^

Although some policy expectations highlight the potential for municipal services to reduce hospital utilization and curtail costs,^38,82^ this assumption may not fully apply to high-cost older patients. Research has shown that their complex healthcare needs often necessitate hospital-based diagnostics, advanced procedures, or intensive care treatment, which cannot be fully substituted by municipal care.^4,83^ Consequently, studies have demonstrated that community and municipal healthcare services do not consistently reduce hospitalizations or associated costs for patients with complex needs.^15,84^ For example, research by Smeets et al^14^ and Pestka et al^15^ indicate that while addressing the complex needs of patients in primary care is critical, even well-developed municipal and primary care services may be insufficient to prevent hospitalization due to the unpredictable and acute nature of the health conditions associated with high-cost older patients. This is further corroborated by Nordic studies, showing that frequent users of municipal healthcare often remain significantly dependent on specialized care.^85,86^

Thus, the role of municipal services for high-cost older patients in Norway may be less about preventing hospital use and more about ensuring safe transitions from hospitals to home, supporting recovery, and managing care complexity outside of hospital settings.^87,88^ Home and transitional care services can help address these needs by providing continuity of care and reducing adverse health outcomes after hospital discharge.^89-91^ However, ensuring continuity of care depends on efficient collaboration between hospitals and municipalities.^40^ Otherwise, the intended cooperation between services may not be fully realized, despite the availability of services.^40,92^ Despite reforms aimed at improving care transitions,^20,46^ studies have shown that challenges in coordination across healthcare levels, such as communication and sharing of responsibility between healthcare sectors in Norway, continue to persist.^25,46^ Additionally, individual preferences, restrictive eligibility criteria and limited service availability in some municipalities may also prevent some patients from fully utilizing municipal healthcare services,^58,93^ further necessitating hospital care.^25,94^

While our findings highlight the importance of municipal care for high-cost older patients, our data limits us from assessing the quality, continuity and integration of these services and coordination between healthcare sectors. Research does however indicate that there currently remains a critical need to strengthen care coordination strategies to enhance the continuity of care, effectiveness, and efficiencies of these services in Norway.^20,25^

### Implications for Future Research Initiatives

Future research should evaluate the quality and adequacy of municipal healthcare services, including home healthcare and short- and long-term care, to better understand their role in meeting the needs of older adults with complex healthcare needs. Comparing the top 10% to more narrowly defined subgroups may also provide other nuanced insights into cost-related gradients. Other studies should also investigate long-term healthcare utilization among high-cost populations, which may reveal new insights into the observed dual dependency on municipal and hospital services. More research is needed to evaluate if the ongoing efforts to strengthen continuity of care and cross-sectoral collaboration are effectively meeting the needs of high-cost older patients in Norway.

### Strength and Limitations

The main strength of this study is its large sample size obtained through registries, which allows for obtaining precise estimates across different age categories and by high-cost status. It is also a major strength that the data is leveraged from three national government-regulated registries which provide comprehensive information on municipal healthcare utilization, specialized healthcare, and sociodemographic characteristics. The large sample size ensures that the study can detect even small differences in service utilization between groups. The use of sensitivity analyses also strengthens the internal validity and reliability of our findings. The study’s alignment with national healthcare priorities strengthens its relevance and underscores its potential contribution to informing ongoing policy and service development efforts in Norway.^22,39,95^ In terms of generalizability, our findings may be transferable to countries with a similar healthcare structure as Norway, or to countries operating with a universal healthcare plan, such as the Beveridge model or national health insurance.^96^

The cross-sectional design restricts the study’s ability to infer causal relationships between the patients’ characteristics and their healthcare utilization. Longitudinal data would have provided a more comprehensive view on municipal healthcare utilization over an extended period, which might further explain some of the observed variations in duration of service use. While the study adjusts for several important individual characteristics, it does not include data on other influential factors, such as lifestyle characteristics (eg, physical activity, diet, alcohol and tobacco use, and drug consumption).^97^

As with any categorization of variables, there is potential for misclassification. For instance, the use of DRG-weights as the sole determinant for defining high-cost status may not fully capture the complexity of healthcare needs among older adults. By using DRG instead of actual cost, we might underestimate the association between high-cost/non-high-cost status and our outcome variables. The actual difference between the groups may therefore be larger than what was observed in our study. As our findings show clear differences between the groups for various outcomes, we argue that the results are robust, and the use of DRG weights, while being a limitation, does not challenge the main conclusions of this study.

Similarly, while excluding deceased participants ensures complete datasets, we risk underestimating the overall service use, particularly for end-of-life care where healthcare needs and service utilization are typically highest.^98^ However, the sensitivity analysis mitigated this limitation, confirming that the findings remain robust despite excluding these participants. Lastly, while the quality of data provided by the NRPHC has reportedly improved in recent years, there may still be instances of misregistration that are not detectable by researchers.^65^ Despite these limitations, the study’s strengths in design and data sources provide valuable insights into the patterns of municipal healthcare utilization among high-cost older patients in Norway.

## Conclusion

This study offers new insights into the utilization of municipal healthcare services among high-cost older patients in Norway. A larger proportion of the high-cost group received municipal healthcare services in 2019. Among those who received services, the high-cost group had longer duration of service use for institutional short-term treatment/examination and rehabilitation/habilitation than the non-high-cost group, but shorter duration for institutional long-term care. The proportions of service use increased with age in both the high-cost and non-high-cost groups. While the high-cost group had greater odds of receiving services across all age categories, the duration of service use varied. Overall, the findings show that high-cost older patients have substantial healthcare needs that extend beyond somatic hospitals into municipal healthcare. The observed service use among high-cost older patients highlights the important role municipal healthcare services play in caring for older adults with complex healthcare needs, underscoring the importance of strengthening the continuity of care, effectiveness and efficiencies of these services in Norway.

## Supplemental Material

sj-docx-1-his-10.1177_11786329251406082 – Supplemental material for Utilization of Municipal Healthcare Services Among High-Cost Older Patients in Norwegian Somatic Hospitals: A Cross-Sectional Registry Study Supplemental material, sj-docx-1-his-10.1177_11786329251406082 for Utilization of Municipal Healthcare Services Among High-Cost Older Patients in Norwegian Somatic Hospitals: A Cross-Sectional Registry Study by Morten Lønhaug-Næss, Monika Dybdahl Jakobsen, Bodil H. Blix, Karl Ove Hufthammer and Jill-Marit Moholt in Health Services Insights
