# Supplementary material for: Utilization of Municipal Healthcare Services Among High-Cost Older Patients in Norwegian Somatic Hospitals: A Cross-Sectional Registry Study
Source: Health Serv Insights. 2025 Dec 23;18:11786329251406082. doi: 10.1177/11786329251406082 (PMC12743808; doi:10.1177/11786329251406082)
Supplement: sj-docx-1-his-10.1177_11786329251406082 – Supplemental material for Utilization of Municipal Healthcare Services Among High-Cost Older Patients in Norwegian Somatic Hospitals: A Cross-Sectional Registry Study [file sj-docx-1-his-10.1177_11786329251406082.docx]

| **Supplemental Table 1** – Overview of included covariates with coding from Statistics Norway (SSB) and the Norwegian Patient Registry (NPR) | | | |
| --- | --- | --- | --- |
| Variable name | Recording | | Recoding |
| **Sex**  *The sex/gender of the patient (binary).*  Registry: *NPR* | a. Men, b. Women | |  |
| *Sex was included to adjust for observed differences in healthcare seeking behaviors, where women typically are more likely to utilize primary healthcare services, whereas men have a higher utilization of specialized services.* | | | |
| **Marital status**  *The marital status of the person across 4 categories, recoded to a binary variable.*  Registry: *SSB* | a. Single, unmarried,  b. Married, registered partner/cohabitant,  c. Separated, Divorced,  d. Widowed | | a. Single, unmarried, separated, divorced, widowed  b. Married, registered partner/cohabitant |
| *Marital status was included due to evidence suggesting that single, widowed or divorced individuals may have fewer caregiving options, and thus rely more on healthcare services.* | | | |
| **Educational level**  *The highest educational attainment across 5 groups, recoded to 4 categories.*  Registry: *SSB* | a. Primary school  b. High school  c. Vocational school  d. University/college short  e. University/college long | | 1. Primary school 2. High school/Vocational school 3. University/college short 4. University/college long |
| *Educational level was included as higher educational attainment has previously been associated with increased health literacy and greater likelihood of engaging in preventive health behaviors.* | | | |
| ***Household income***  *Income after tax across 8 categories. Defined by SSB as: professional income, capital income, taxable and tax-free transfers during the calendar year.*  Registry: *SSB* | a. < 150,000 NOK  b. 150-250,000 NOK  c. 251-350, 000 NOK  d. 351-450,000 NOK  e. 451-550,000 NOK  f. 551-750,000 NOK  g. 751-1000,000 NOK  h. > 1000,000 NOK | |  |
| *Household income was included as income status is a well-documented determinant of healthcare utilization, with lower income often linked to increased use of healthcare services.* | | | |
| **Index of municipal centrality**  *Index of municipal centrality across 6 categories. The index measures the distance to workplaces and various services, including healthcare services such as hospitals.*  Registry: *SSB* | a. Most central  b. Second most central  c. Mid-central I  d. Mid-central II  e. Second least central  f. Least central | |  |
| *The index of municipal centrality was included as more central municipalities typically have better infrastructure in terms of inhabitants, workplaces, and centralized healthcare facilities such as hospitals. The availability of municipal services may vary based on the urban or rural setting of the residents.* | | | |
| **Main Diagnosis Groups**  *The number of different main diagnoses groups are derived from the 22 available main diagnosis from NPR, recoded into 5 categories based on the number of different main diagnoses. Main diagnoses registered with a patient during admission to Norwegian Somatic hospitals.*  Registry: *NPR* | - Diseases of the nervous system - Diseases in breast tissue - Diseases of the female genitalia - Ear, nose, and throat disorders - Diseases of the respiratory organs - Diseases of the circulatory organs - Diseases of the blood, blood-forming organs, and immune system - Diseases of the digestive organs - Myeloproliferative disorders and differentiated tumors - Categories across multiple diagnostic groups - Infections and parasitic diseases - Burns and burn related wounds - Categories for error and uncommon diagnosis procedure combinations | - Diseases of the liver, biliary tract, and pancreas - Mental disorders and substance abuse problems - Diseases of the musculoskeletal system and connective tissue - Trauma, poisoning and toxic effect of drugs/other substances, drug abuse and organic mental disorders - Diseases of the skin and subcutaneous tissue - Internal secretory, nutritional, and metabolic disorders - Kidney and urinary tract disorders - Diseases of the male genitalia | 1. 1 main diagnosis 2. 2 different main diagnosis 3. 3 different main diagnosis 4. 4 different main diagnosis 5. 5 or more different main diagnosis |
| *The number of main diagnosis groups were included as a proxy measure for multimorbidity, reflecting the number of registered different main diagnoses across the organ systems.* | | | |

| **Supplemental Table 2**: Unadjusted regression models for the likelihood of receiving services and duration (hours/days) of service use among high-cost older patient compared to non-high-cost older patients in the study sample, stratified by age. | | | | | | | | | | | | | | |
| --- | --- | --- | --- | --- | --- | --- | --- | --- | --- | --- | --- | --- | --- | --- |
|  | Unadjusted bivariate regression  (*n* = 189,336) ^a^ | | | Unadjusted gamma regression with log link  (*n* = 61,171) ^b^ | | | | | | | | | | |
|  | OR | *P-*value | 95 % CI | | *n* | exp *β* | | *P-*value | | | | 95% CI | | |
| *Home healthcare services ^*^* | | | |  | | | | | | | | | | |
| 65-74 years | 4.94 | < 0.001 | 4.70–5.19 | | *11,211* | 0.91 | | 0.061 | | | | 0.82–1.01 | | |
| 75-84 years | 2.87 | < 0.001 | 2.72–3.01 | | *18,257* | 1.01 | | 0.805 | | | | 0.93–1.10 | | |
| ≥ 85 years | 2.07 | < 0.001 | 1.91–2.24 | | *18,984* | 1.05 | | 0.200 | | | | 0.97–1.14 | | |
| All age categories | 2.72 | < 0.001 | 2.64–2.81 | | *48,452* | 0.94 | | 0.008 | | | | 0.89–0.98 | | |
| *Municipal acute bed units ^**^* | | | |  | | | | | | | | | | |
| 65-74 years | 2.49 | < 0.001 | 2.19–2.83 | | *1,373* | 1.42 | | 0.016 | | | | 1.07–1.90 | | |
| 75-84 years | 1.61 | < 0.001 | 1.44–1.79 | | *2,506* | 1.22 | | 0.194 | | | | 0.91–1.63 | | |
| ≥ 85 years | 1.30 | < 0.001 | 1.15–1.48 | | *3,373* | 1.05 | | 0.740 | | | | 0.77–1.41 | | |
| All age categories | 1.48 | < 0.001 | 1.37–1.58 | | *7,252* | 1.18 | | 0.060 | | | | 0.99–1.39 | | |
| *Institutional short-term care: treatment/examination ^**^* | | | |  | | |  | | |  | | |  |  |
| 65-74 years | 4.49 | < 0.001 | 4.14–4.88 | | *2,763* | 1.18 | | < 0.001 | | | | 1.08–1.30 | | |
| 75-84 years | 2.85 | < 0.001 | 2.66–3.05 | | *5,613* | 1.10 | | 0.007 | | | | 1.03–1.18 | | |
| ≥ 85 years | 2.21 | < 0.001 | 2.03–2.41 | | *7,354* | 1.24 | | < 0.001 | | | | 1.15–1.33 | | |
| All age categories | 2.46 | < 0.001 | 2.36–2.57 | | *15,730* | 1.14 | | < 0.001 | | | | 1.09–1.19 | | |
| *Institutional short-term care: rehabilitation/habilitation ^**^* | | | |  | | | | |  | | | | | |
| 65-74 years | 4.75 | < 0.001 | 4.34–5.21 | | *2,205* | 1.25 | | < 0.001 | | | | 1.13–1.38 | | |
| 75-84 years | 3.44 | < 0.001 | 3.19–3.71 | | *3,783* | 1.22 | | < 0.001 | | | | 1.14–1.30 | | |
| ≥ 85 years | 2.84 | < 0.001 | 2.59–3.13 | | *4,078* | 1.33 | | < 0.001 | | | | 1.22–1.44 | | |
| All age categories | 3.13 | < 0.001 | 2.94–3.29 | | *10,066* | 1.24 | | < 0.001 | | | | 1.19–1.30 | | |
| *Institutional long-term care ^**^* | | | |  | | | |  | | |  | | | |
| 65-74 years | 1.87 | < 0.001 | 1.59–2.21 | | *1,013* | 0.72 | | < 0.001 | | | | 0.64–0.80 | | |
| 75-84 years | 1.16 | 0.010 | 1.04–1.31 | | *2,849* | 0.65 | | < 0.001 | | | | 0.60–0.71 | | |
| ≥ 85 years | 1.05 | 0.369 | 0.94–1.18 | | *5.142* | 0.69 | | < 0.001 | | | | 0.64–0.74 | | |
| All age categories | 1.01 | 0.731 | 0.94–1.09 | | *9.004* | 0.68 | | < 0.001 | | | | 0.65–0.72 | | |
| ^a^ Unadjusted bivariate regression for the likelihood of receiving the various municipal healthcare services for the high-cost group compared to the non-high-cost group in the study sample (*n* = 189,336), stratified by age. - reports the OR for the likelihood for the high-cost group to receive the included municipal healthcare services compared to the non-high-cost group.  *OR* (odds ratio), *CI* (confidence interval).  ^b^ Unadjusted gamma regression for the duration (hours/days) of service use of municipal healthcare services among the high-cost group compared to the non-high-cost group for those participants who received services (*n* = 61,171), stratified by age in 2019.  Exp *β*: Reported coefficient for the high-cost group.  * Measured in hours of services received. ** Measured in days admitted. | | | | | | | | | | | | | | |

| **Supplemental Table 3:** Overview of mean duration of service use of municipal healthcare services for high-cost and non-high-cost older patients who received each of the municipal healthcare services in 2019 (n = 61,171), by age. | | | | | | | | |
| --- | --- | --- | --- | --- | --- | --- | --- | --- |
|  | | High-cost older patients | | | Non-high-cost older patients | | *P*-value | |
|  |  | | *n* | mean (SD), 95% CI | *n* | mean (SD), 95% CI | *t*-test *^a^* | MW test *^b^* |
| *Home healthcare services^*^* | | | | |  |  |  |  |
| 65-74 years | | | *3,314* | 132 (326) 121–143 | *7,897* | 145 (381) 137–154 | 0.057 | 0.907 |
| 75-84 years | | | *3,503* | 138 (317) 127–148 | *14,754* | 136 (265) 132–141 | 0.806 | 0.182 |
| ≥ 85 years | | | *1,777* | 132 (326) 121–143 | *17,207* | 145 (381) 137–154 | 0.210 | 0.237 |
| All age categories | | | *8,594* | 146 (319) 139–152 | *39,858* | 155 (303) 153–159 | 0.007 | < 0.001 |
| *Municipal acute bed units^**^* | | | | |  |  |  |  |
| 65-74 years | | | *312* | 8 (20) 6–11 | *1,061* | 6 (10) 5–6 | 0.037 | 0.145 |
| 75-84 years | | | *400* | 8 (23) 6–10 | *2,106* | 7 (14) 6–7 | 0.232 | 0.893 |
| ≥ 85 years | | | *295* | 8 (20) 5–10 | *3,078* | 7 (12) 7–8 | 0.747 | 0.725 |
| All age categories | | | *1,007* | 8 (21) 7–9 | *6,245* | 7 (13)7–7 | 0.080 | 0.455 |
| *Institutional short-term care: treatment/examination^**^* | | | | |  |  |  |  |
| 65-74 years | | | *919* | 34 (38) 32–37 | *1,844* | 29 (37) 27–31 | < 0.001 | < 0.001 |
| 75-84 years | | | *1,308* | 35 (39) 33–37 | *4,305* | 32 (38) 30–33 | 0.008 | < 0.001 |
| ≥ 85 years | | | *886* | 41 (41) 38–44 | *6,468* | 33 (35) 32–34 | < 0.001 | < 0.001 |
| All age categories | | | *3,113* | 37 (39) 35–38 | *12,617* | 32 (36) 31–33 | < 0.001 | < 0.001 |
| *Institutional short-term care: rehabilitation/habilitation^**^* | | | | |  |  |  |  |
| 65-74 years | | | *768* | 38 (43) 35–41 | *1,437* | 30 (35) 28–32 | < 0.001 | < 0.001 |
| 75-84 years | | | *1,029* | 36 (34) 34–38 | *2,754* | 29 (30) 28–30 | < 0.001 | < 0.001 |
| ≥ 85 years | | | *629* | 42 (41) 39–45 | *3,449* | 32 (31) 31–33 | < 0.001 | < 0.001 |
| All age categories | | | *2,426* | 38 (39) 36–39 | *7,640* | 31 (32) 30–31 | < 0.001 | < 0.001 |
| ***Institutional long-term care^**^*** | | | | |  |  |  |  |
| 65-74 years | | | *184* | 157 (111) 141–173 | *829* | 220 (126) 211–228 | < 0.001 | < 0.001 |
| 75-84 years | | | *349* | 144 (109) 133–156 | *2,500* | 221 (127) 216–226 | < 0.001 | < 0.001 |
| ≥ 85 years | | | *376* | 149 (112) 138–161 | *4,766* | 216 (127) 213–221 | < 0.001 | < 0.001 |
| All age categories | | | *909* | 149 (111) 142–156 | *8,095* | 219 (127) 216–221 | < 0.001 | < 0.001 |
| *n* (number of participants who received each service type), *SD* (standard deviation), *CI* (confidence interval).  * Measured in hours of services received. ** Measured in days admitted.  ^a^ two-sided *p*-value for Welch’s t-test, ^b^ *p*-value for the Mann-Whitney U test | | | | | | | | |

| **Supplemental Table 4:** Sensitivity analysis for the duration (hours/days) of service use of municipal healthcare services among high-cost older patients compared to non-high-cost older patients who received services, excluding participants with services use exceed 8760 hours/365 days (*n* = 349) by age (*n* = 60,822). | | | | | | |
| --- | --- | --- | --- | --- | --- | --- |
|  | Unadjusted ^a^ | | | Adjusted ^b^ | | |
|  | exp *β* | *P-*value | 95% CI | exp *β* | *P*-value | 95% CI |
| *Home healthcare services ^*^* | | | |  |  |  |
| 65-74 years | 0.91 | 0.079 | 0.83–1.01 | 0.89 | 0.042 | 0.80–0.99 |
| 75-84 years | 0.99 | 0.695 | 0.92–1.06 | 0.99 | 0.719 | 0.92–1.06 |
| ≥ 85 years | 1.05 | 0.209 | 0.97–1.14 | 1.11 | 0.023 | 1.01–1.21 |
| All age categories | 0.93 | 0.002 | 0.88–0.97 | 0.97 | 0.198 | 0.92–1.02 |
| *Municipal acute bed units ^**^* | | | |  |  |  |
| 65-74 years | 1.42 | 0.016 | 1.07–1.90 | 1.24 | 0.036 | 1.10–1.51 |
| 75-84 years | 1.22 | 0.195 | 0.91–1.63 | 1.16 | 0.232 | 0.91–1.49 |
| ≥ 85 years | 1.05 | 0.726 | 0.78–1.41 | 1.05 | 0.714 | 0.81–1.36 |
| All age categories | 1.00 | 0.941 | 0.90–1.12 | 1.15 | 0.094 | 0.98–1.36 |
| *Institutional short-term care: treatment/examination^**^* | | | |  |  |  |
| 65-74 years | 1.18 | < 0.001 | 1.08–1.29 | 1.15 | 0.004 | 1.05–1.26 |
| 75-84 years | 1.10 | < 0.001 | 1.02–1.17 | 1.06 | 0.132 | 0.98–1.13 |
| ≥ 85 years | 1.24 | < 0.001 | 1.16–1.33 | 1.21 | < 0.001 | 1.13–1.31 |
| All age categories | 1.13 | < 0.001 | 1.09–1.18 | 1.12 | < 0.001 | 1.07–1.17 |
| *Institutional short-term care: rehabilitation/habilitation^**^* | | | |  |  |  |
| 65-74 years | 1.22 | < 0.001 | 1.12–1.34 | 1.14 | 0.007 | 1.04–1.26 |
| 75-84 years | 1.22 | < 0.001 | 1.14–1.30 | 1.18 | < 0.001 | 1.09–1.27 |
| ≥ 85 years | 1.32 | < 0.001 | 1.22–1.43 | 1.29 | < 0.001 | 1.19–1.41 |
| All age categories | 1.23 | < 0.001 | 1.18–1.29 | 1.20 | < 0.001 | 1.14–1.26 |
| *Institutional long-term care^**^* | | | |  |  |  |
| 65-74 years | 0.72 | < 0.001 | 0.64–0.80 | 0.78 | < 0.001 | 0.68–0.89 |
| 75-84 years | 0.67 | < 0.001 | 0.62–0.73 | 0.80 | < 0.001 | 0.73–0.88 |
| ≥ 85 years | 0.71 | < 0.001 | 0.65–0.76 | 0.82 | < 0.001 | 0.75–0.89 |
| All age categories | 0.70 | < 0.001 | 0.66–0.73 | 0.82 | < 0.001 | 0.77–0.86 |
| Gamma regression with log link for municipal healthcare services among high-cost and non-high-cost older patients, stratified by age in 2019. * Measured in hours of services received. ** Measured in days admitted. Exp *β*: Reported coefficient for the high-cost group.  ^a^ Reporting only the covariate for high-cost status. ^b^ The model is adjusted for: Sex, marital status, educational level, household income, index of municipal centrality, and number of main diagnoses. | | | | | | |

| **Supplemental Table 5:** Sensitivity analysis for the study population (*n* = 211,738) comparing high-cost older patients to non-high-cost older patients on the impact of including deceased participants (*n* = 22.402) on the binary logistic and gamma regression models, adjusted for sociodemographic and clinical factors, stratified by age. | | | | |
| --- | --- | --- | --- | --- |
|  | Binary logistic regression ^a^ | | Gamma regression ^b^ | |
|  | OR (95% CI) | *P-*value | exp *β* (95% CI) | *P-*value |
| *Home healthcare services ^*^* | | |  |  |
| - 1. years | 3.56 (3.39–3.80) | < 0.001 | 0.88 (0.79–0.98) | 0.024 |
| 75-84 years | 2.38 (2.26–2.52) | < 0.001 | 1.00 (0.93–1.08) | 0.967 |
| ≥ 85 years | 1.68 (1.54–1.83) | < 0.001 | 1.07 (1.01–1.13) | 0.027 |
| All age categories | 2.36 (2.29–2.45) | < 0.001 | 0.97 (0.92–1.02) | 0.222 |
| *Municipal acute bed units ^**^* | | |  |  |
| 65-74 years | 1.60 (1.39–1.85) | < 0.001 | 1.24 (1.02–1.52) | 0.035 |
| 75-84 years | 1.23 (1.09–1.38) | < 0.001 | 1.16 (0.91–1.49) | 0.233 |
| ≥ 85 years | 1.04 (0.91–1.19) | 0.559 | 1.04 (0.81–1.36) | 0.745 |
| All age categories | 1.17 (1.09–1.26) | < 0.001 | 1.15 (0.97–1.36) | 0.098 |
| *Institutional short-term care: treatment/examination^**^* | | |  |  |
| 65-74 years | 3.36 (3.06–3.69) | < 0.001 | 1.15 (1.05–1.28) | 0.005 |
| 75-84 years | 2.50 (2.32–2.69) | < 0.001 | 1.06 (0.98–1.13) | 0.138 |
| ≥ 85 years | 1.91 (1.75–2.09) | < 0.001 | 1.21 (1.12–1.30) | < 0.001 |
| All age categories | 2.23 (2.13–2.34) | < 0.001 | 1.12 (1.07–1.17) | < 0.001 |
| *Institutional short-term care: rehabilitation/habilitation^**^* | | |  |  |
| 65-74 years | 3.55 (3.21–3.93) | < 0.001 | 1.16 (1.05–1.28) | 0.004 |
| 75-84 years | 2.94 (2.70–3.19) | < 0.001 | 1.18 (1.09–1.27) | < 0.001 |
| ≥ 85 years | 2.48 (2.23–2.75) | < 0.001 | 1.30 (1.19–1.42) | < 0.001 |
| All age categories | 2.74 (2.60–2.89) | < 0.001 | 1.21 (1.15–1.27) | < 0.001 |
| *Institutional long-term care^**^* | | |  |  |
| 65-74 years | 1.77 (1.48–2.12) | < 0.001 | 0.73 (0.65–0.83) | < 0.001 |
| 75-84 years | 1.38 (1.22–1.57) | < 0.001 | 0.77 (0.67–0.87) | < 0.001 |
| ≥ 85 years | 1.32 (1.17–1.49) | < 0.001 | 0.80 (0.73–0.86) | < 0.001 |
| All age categories | 1.27 (1.18–1.37) | < 0.001 | 0.80 (0.76–0.84) | < 0.001 |
| The model is adjusted for: Sex ^a^, marital status ^b^, educational level ^c^, index of municipal centrality ^d^, and number of main diagnoses ^e^  ^a^ Binomial logistic regression model - reports the OR for the likelihood for the high-cost group to receive the included municipal healthcare services compared to the non-high-cost group. Includes all participants (alive and dead) in the sample size.  *OR* (odds ratio), *CI* (confidence interval).  ^b^ Gamma regression with log link for municipal healthcare services among high-cost and non-high-cost older patients, stratified by age category in 2019. Includes all participants (alive and dead) who received services in 2019. Exp *β*: Reported exponentiated coefficient for high-cost older patients  * Measured in hours of services received. ** Measured in days admitted. Reported coefficient for high-cost older patients. | | | | |
